# Measuring stress signaling responses of stomata in isolated epidermis of graminaceous species

**DOI:** 10.3389/fpls.2015.00533

**Published:** 2015-07-13

**Authors:** Lei Shen, Peng Sun, Verity C. Bonnell, Keith J. Edwards, Alistair M. Hetherington, Martin R. McAinsh, Michael R. Roberts

**Affiliations:** ^1^Lancaster Environment Centre, Lancaster University, Lancaster, UK; ^2^School of Biological Sciences, University of Bristol, Bristol, UK

**Keywords:** stomata, guard cells, isolated epidermis, cereal, Gramineae, abscisic acid, carbon dioxide, temperature

## Abstract

Our current understanding of guard cell signaling pathways is derived from studies in a small number of model species. The ability to study stomatal responses in isolated epidermis has been an important factor in elucidating the mechanisms by which the stomata of these species respond to environmental stresses. However, such approaches have rarely been applied to study guard cell signaling in the stomata of graminaceous species (including many of the world’s major crops), in which the guard cells have a markedly different morphology to those in other plants. Our understanding of guard cell signaling in these important species is therefore much more limited. Here, we describe a procedure for the isolation of abaxial epidermal peels from barley, wheat and *Brachypodium distachyon*. We show that isolated epidermis from these species contains viable guard cells that exhibit typical responses to abscisic acid (ABA) and CO_2_, as determined by measurements of stomatal apertures. We use the epidermal peel assay technique to investigate in more detail interactions between different environmental factors in barley guard cells, and demonstrate that stomatal closure in response to external CO_2_ is inhibited at higher temperatures, whilst sensitivity to ABA is enhanced at 30°C compared to 20 and 40°C.

## Introduction

The maintenance of global food is one of the greatest challenges currently facing plant scientists. Water availability is a major constraint on crop yield (Sinclair and Rufty, 2012) and is the single most important factor limiting food production, with significant yield losses reported under water deficit (Boyer, 1982; Mueller et al., 2012; van Ittersum et al., 2013). Stomata play a key role in determining crop water use efficiency (biomass production or yield per unit of water used), through the regulation of the exchange of water vapor and CO_2_ between plant tissues and the atmosphere (Mansfield et al., 1990; Hetherington and Woodward, 2003; Yoo et al., 2009). This gaseous exchange is controlled by the size of the stomatal pore, which is determined by changes in the turgor of the pair of specialized guard cells that surround the pore and which in turn are driven by fluxes of anions and cations (Pandey et al., 2007; Kim et al., 2010; Hedrich, 2012; Kollist et al., 2014). Guard cells integrate information from a variety of internal and external environmental signals in order to formulate the optimal pore size for a given set of environmental conditions (Mansfield et al., 1990; Hetherington and Woodward, 2003; Kim et al., 2010). For example, stomata close in response to abscisic acid (ABA), produced under conditions of limited water availability, and to elevated CO_2_ (Mansfield et al., 1990; Hetherington and Woodward, 2003; Kim et al., 2010). In contrast, stomata open at low CO_2_ concentrations (Bunce, 2007), in high light (Shimazaki et al., 2007) and in response to auxin (Acharya and Assmann, 2009). Guard cells also respond to other environmental signals, such as the atmospheric pollutant ozone (Vainonen and Kangasjarvi, 2015) and pathogenic microbes (Sawinski et al., 2013), resulting in stomatal closure and thereby preventing entry to the leaf of damaging chemical and biological agents.

The diversity of stimuli to which stomata respond, together with the ease with which the response can be quantified (i.e., changes in stomatal aperture or conductance), have meant that guard cells have been extensively used as a model system for studying signaling pathways in plant cells (Mansfield et al., 1990; Hetherington and Brownlee, 2004; Kim et al., 2010). This has resulted in the elucidation of a complex signaling network controlling the molecular machinery integrating the different signals to which guard cells are exposed in order to regulate guard cell turgor (Mansfield et al., 1990; Hetherington and Woodward, 2003; Kim et al., 2010). The ability to measure changes in stomatal aperture in isolated epidermis, in response to externally-applied signals, and to manipulate these responses both pharmacologically and genetically, has been central to the advances in understanding of guard cell signaling that have been made in the last 20 years.

To date, studies of guard cell signaling have focused on a small number of model species, notably *Vicia faba*, *Commelina communis* and latterly, *Arabidopsis thaliana* (for reviews, see Hetherington and Brownlee, 2004; Kim et al., 2010). The stomata of all of these species possess kidney bean-shaped guard cells, which are typical of the large majority of plant families, including the mosses, ferns, gymnosperms and most angiosperms (Willmer and Fricker, 1996). However, the stomata of the monocotyledonous family, Gramineae (Poaceae; the true grasses), which includes the world’s major cereal crops, have a different morphology, possessing characteristic dumb-bell shape guard cells and a pair of specialized subsidiary cells. The different morphology of graminaceous stomata provide them with different mechanical properties, which likely allow them to open and close more rapidly in response to environmental signals (Franks and Farquhar, 2007). It is therefore critical to understand fully the molecular mechanisms by which the stomata of the graminaceous species respond to environmental stresses, particularly in relation to the protection of global food security and the challenge of producing “more crop per drop” (Kijne et al., 2003) posed by future environmental changes in global temperature, CO_2_ levels and water availability.

Although assays of stomatal responses have been performed using isolated epidermis from maize and wild grasses (Pallaghy, 1971; Incoll and Whitelam, 1977; Jewer and Incoll, 1980; Rodriguez and Davies, 1982), in general, graminaceous species are commonly regarded as poorly tractable systems for epidermal peel isolation. Other authors have isolated epidermal tissue for other purposes, such as microscopy (Zou et al., 2011) or metabolite analysis (Falter et al., 2015), but in these cases, tissues were not demonstrated to be suitable for stomatal assays. Here, we demonstrate that the epidermal peel assay used so extensively in other model systems is also applicable to model grass species, and we use it to identify interactions between temperature and signals stimulating stomatal closure in barley.

## Materials and Methods

### Plant Material

Seeds of *Brachypodium distachyon* (line *Bd21,* Vogel et al., 2006) were sown in a 1:1 mix of Sinclair multipurpose compost and silver sand (Sinclair Horticultural, UK) and grown under a 16-h photoperiod at 22°C ± 2°C, 70% relative humidity and 120 μmol m^–2^ s^–1^ photosynthetic photon flux density (PPFD) in a Microclima growth cabinet (CEC, Glasgow, UK). Wheat (cultivar Cadenza) and barley (cultivars Golden Promise and Optic) were grown in Levington M3 peat-based compost in a heated, passively ventilated glasshouse (minimum temperature 15°C, mean day-time temperature 25°C) with 14 h of supplementary lighting supplied by 125 W 50/60 Hz High output, Correct Spectrum Class 11 energy saving bulbs (wheat) or Osram Greenpower 600 W high pressure sodium lamps (barley). 2 days prior to peeling, plants were moved to controlled environment chambers set at 22°C, with a photoperiod of 14 h (wheat) or 16 h (barley) with Osram fluora lamps delivering 70–100 μmol m^–2^ s^–1^ light.

### Preparation of Isolated Epidermis

**FIGURE 1:**
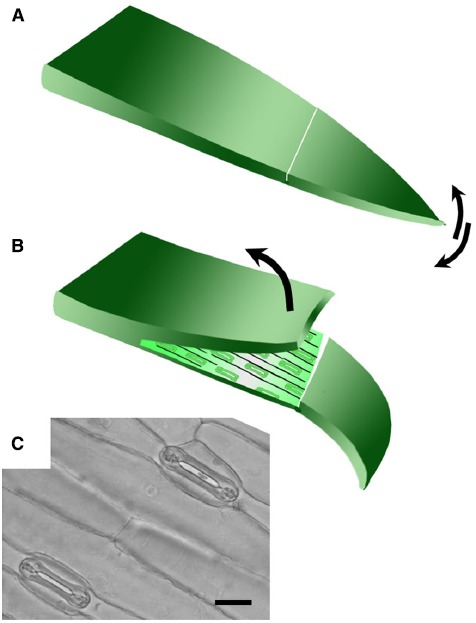
**Technique for removing abaxial epidermis. (A)** The first true leaf is removed from the plant, bent over the finger adaxial side up, and a cut made across the lamina using a scalpel blade. The tip of the leaf blade is bent back and forth to detach a small section of the mesophyll from the lower epidermis. **(B)** The upper layer was then peeled back using forceps to leave the lower epidermis attached to the leaf tip. The size of the epidermal peel obtained typically varies from around 1–3 cm. **(C)** Bright field micrograph illustrating open stomata in a typical abaxial epidermal peel from wheat (20 μm scale bar).

Isolated epidermis was obtained from the abaxial surface of the first true leaf of 8–14 day old wheat and barley plants, when the first true leaf had stopped expanding (5–8 cm long). For *Brachypodium distachyon*, the youngest fully expanded leaves of 3–4 week old plants were used. Leaves were cut from the plant and bent over the forefinger with the adaxial surface facing upward. A shallow cut was made with a sharp razor blade horizontally across the leaf and a flap of leaf tissue lifted with a razor, leaving the lower epidermis intact (Figure 1). The leaf tissue was removed from the epidermis with forceps. Once a section of epidermis approximately 1 cm long was exposed, it was cut from the leaf and floated cuticle-side-up in 10 mM MES/KOH (pH 6.2), 50 mM KCl.

### Viability Staining

Isolated epidermis was floated cuticle-side-up in 10 mM MES/KOH (pH 6.2), 50 mM KCl and incubated in a water bath at 22°C at a PPFD of 50–100 μmol m^–2^ s^–1^ provided by an array of five fluorescent tube lights (Sylvania White F13W) underneath the tank. Pieces of epidermis were transferred at intervals to a 0.001% (w/v) solution of fluorescein diacetate (FDA) in 10 mM MES/KOH (pH 6.2), 50 mM KCl and incubated for 30 min prior to observation under the fluorescence microscope (McAinsh et al., 1996). The percentage of viable guard cells was determined by comparing the fluorescent images to bright-field images.

### Promotion of Stomatal Closure Assays

Isolated epidermis was floated cuticle-side-up in Petri dishes containing 10 ml of 10 mM MES/KOH (pH 6.2), 50 mM KCl (a standard buffer to promote stomatal opening; McAinsh et al., 1991) under the conditions described above. Microlances, inserted through small holes in the lids, were used to deliver CO_2_-free air to the Petri dishes. Pieces of epidermis were incubated for 2 h to promote stomatal opening, following which, they were transferred to fresh buffer containing the appropriate concentrations of ABA and incubated for a further one (barley) or two (wheat, *Brachypodium*) hours. ABA was diluted from a 10 mM stock of ( ± )-*cis*, *trans*-ABA dissolved in ethanol. Control solutions lacking ABA always contained ethanol equivalent to the concentration of the highest ABA concentration used. For experiments where CO_2_ concentration was varied, gas from a balanced CO_2_-air cylinder (BOC Industrial Gases, UK) at the appropriate concentration was bubbled through the incubation medium.

### Measurement of Stomatal Apertures

Stomatal apertures were measured at the end of the incubation period by mounting pieces of isolated epidermis onto a microscope slide in a drop of assay buffer with a coverslip. Measurements of stomatal apertures were made using an inverted microscope connected to a sideport-mounted video monitor. A calibrated scale was used to make measurements of the width of the stomatal pore directly from the screen. Stomata containing non-viable guard cells (identifiable by FDA staining) were typically fully closed. Fully closed stomata were therefore not used for measurements. Where comparisons of multiple variables measured in experiments conducted at different times were required, stomatal apertures were expressed as relative values. Relative stomatal aperture was defined as the ratio of stomatal aperture measured under a treatment variable to that measured from the relevant control group.

## Results

### Guard Cells from Wheat, Barley, and *Brachypodium* Remain Viable in Epidermal Peels

**FIGURE 2:**
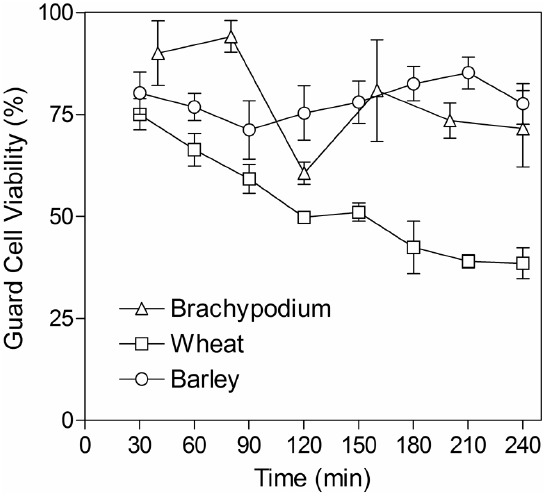
**Guard cell viability in epidermal peels.** Peels were incubated in CO_2_-free MES-KCl buffer at 22°C and then stained for 30 min in FDA. The x-axis indicates the time from the start of the initial incubation period until microscopic observation of FDA staining. Data represent counts from three areas of approximately 1 mm^2^ from each piece of epidermis and three independent biological replicates.

Although grasses are less amenable than current model species used for stomatal research, we developed an approach that could be used routinely to generate intact abaxial epidermis isolated from leaves of wheat, barley and *Brachypodium* seedlings (see “Materials and Methods”). Peels were free of mesophyll cells and contained viable guard cells, subsidiary cells and pavement cells, as determined by FDA staining. Stomata were significantly larger in barley and wheat (typically around 25–50 μm in length) compared to those in the smaller *Brachypodium* plants (guard cells typically 6–9 μm in length). In order to be useful for measuring guard cell-mediated stomatal responses via an *in vitro* assay, it is essential that guard cell viability is maintained in isolated epidermis. We monitored viability over the period when assays are typically performed by performing FDA staining at regular intervals for up to 4 h following isolation. Figure 2 illustrates that guard cell viability was around 80% 30 min after isolation for all species. This level was maintained throughout the test period for both barley and *Brachypodium*, whilst viability gradually declined for wheat.

### Cereal Leaf Epidermis Demonstrates Stomatal Closure in Response to ABA and CO_2_

**FIGURE 3:**
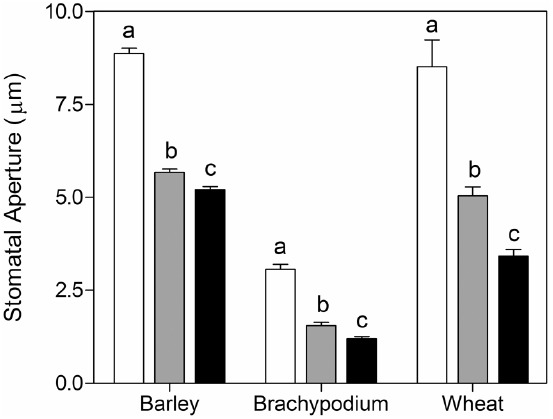
**Promotion of closure of cereal stomata by ABA.** Following incubation under opening conditions, epidermal peels were exposed to zero (white bars), 10^–7^ M (gray bars), or 10^–6^ M (black bars) ABA at 20°C. Values shown are mean stomatal apertures ± SE from *n* = 240 (barley), *n* = 90 (wheat), and *n* = 120 (*Brachypodium*) measurements. Letters indicate statistically different means within species, determined using one-way ANOVA and a Tukey post-test.

**FIGURE 4:**
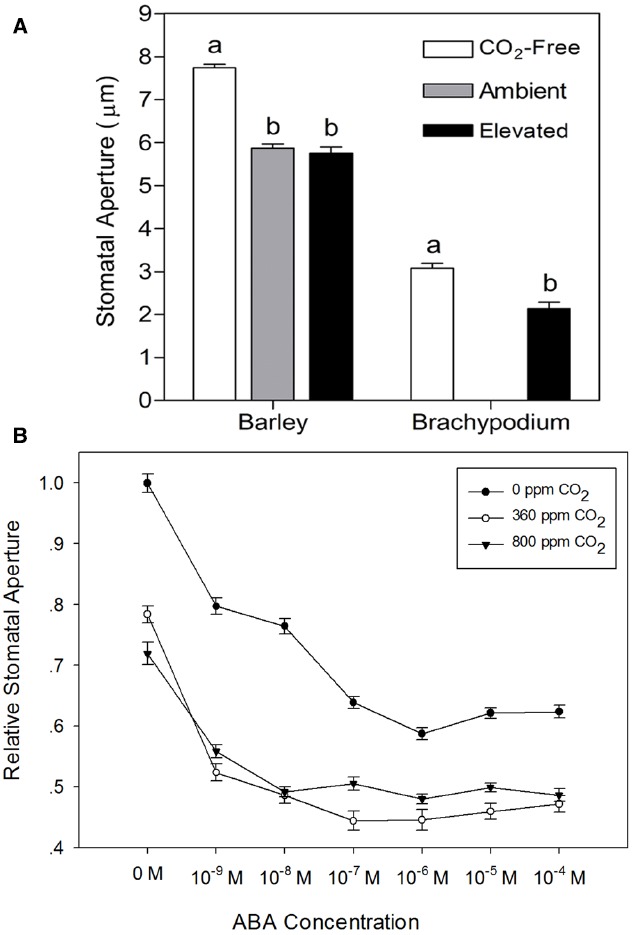
**Responses of cereal stomata to external CO_2_. (A)** Following incubation under opening conditions, epidermal peels were exposed to CO_2_-free air (white bars), or air with ambient CO_2_ (gray bar; 360 ppm, barley), or elevated CO_2_ (black bars; 800 ppm, barley; 700 ppm, *Brachypodium*) at 20°C. Values shown are mean stomatal apertures ± SE (*n* = 120). Letters indicate statistically different means within species, determined using one-way ANOVA with Tukey post-test and a Student’s *t*-test for barley and *Brachypodium* data respectively. **(B)** Interaction between CO_2_ and ABA. Following incubation under opening conditions, barley epidermal peels were exposed to ABA at the concentrations shown on the x-axis under either CO_2_-free air (filled circles), or air with ambient (open circles) or elevated CO_2_ (filled triangles) at 20°C. Values shown are mean stomatal apertures ± SE (*n* = 240).

To test the validity of the stomatal assay in isolated epidermis of graminaceous plants, we examined the well-known response of guard cells to ABA in a promotion of closure assay. As expected, we observed that wheat, barley and *Brachypodium* all exhibit a characteristic dose-dependent response to ABA in the epidermal peel assay (Figure 3). We also measured responses to external CO_2_. In comparison with CO_2_-free air, stomatal closure was promoted by ambient CO_2_ (360 ppm) in barley, but we observed no further response at elevated CO_2_ (800 ppm; Figure 4A). *Brachypodium* stomatal apertures were also reduced by CO_2_ (Figure 4A). We next examined the interaction between ABA and CO_2_ signaling in barley guard cells. We observed a clear additive effect of ABA and CO_2_, with lower stomatal apertures at all concentrations of ABA in the presence of either ambient or elevated CO_2_ relative to CO_2_-free controls (Figure 4B).

### Barley Responses to ABA and CO_2_ are Modified by Elevated Temperature

**FIGURE 5:**
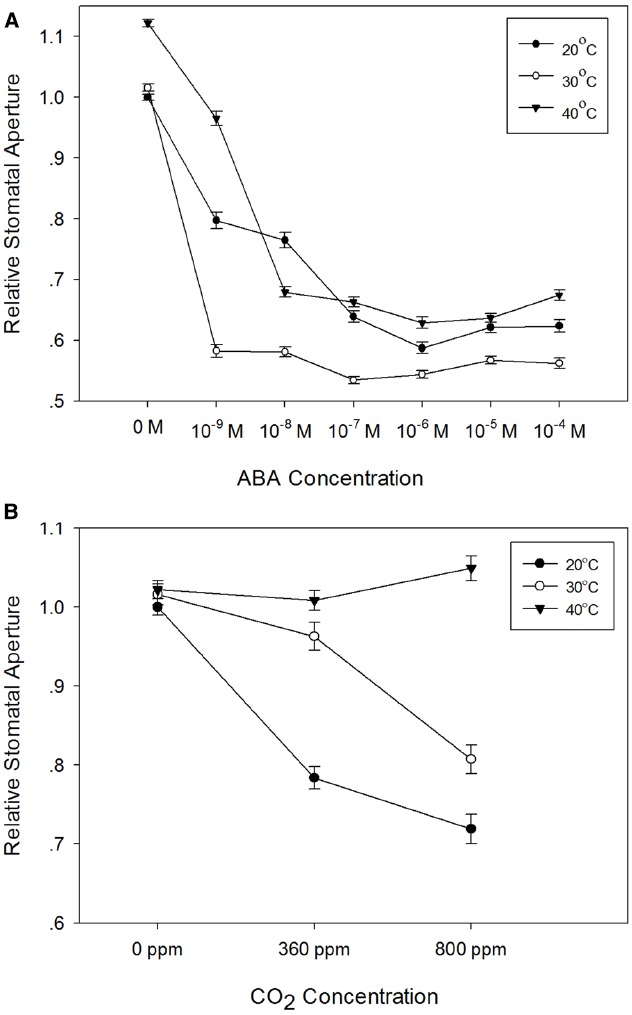
**Temperature-dependence of the responses of barley stomata to ABA and CO_2_. (A)** Interaction between temperature and ABA. Promotion of closure assays were performed at a range of concentrations of ABA in CO_2_-free air at either 20°C (filled circles), 30°C (open circles), or 40°C (filled triangles). To normalize for variation between different experiments, stomatal apertures are expressed relative to that of the control (stomatal aperture at 20°C, no ABA). Data from at least 3 sets of independent experiments were pooled and values are means of at least 120 measurements ± SE. **(B)** Interaction between temperature and CO_2_. Promotion of closure assays were performed in CO_2_-free air or at ambient or elevated CO_2_ at either 20°C (filled circles), 30°C (open circles), or 40°C (filled triangles). To normalize for variation between different experiments, stomatal apertures are expressed relative to that of the control (stomatal aperture at 20°C, no CO_2_). Data from at least three sets of independent experiments were pooled and values are means of at least 120 measurements ± SE.

Stomatal responses to ABA and CO_2_ have been shown to be temperature-dependent (e.g., Raschke, 1970; Rodriguez and Davies, 1982; Spence et al., 1984; Honour et al., 1995). We therefore used the epidermal peel assay to examine the temperature-dependence of ABA- and CO_2_-induced stomatal closure in isolated epidermis of barley. First, we generated dose-response curves for ABA in isolated epidermis incubated at 20, 30, or 40°C. The results presented in Figure 5A, show that in comparison with the response at 20°C, incubation at 30°C significantly increased the sensitivity of guard cells to ABA. Increasing the temperature to 30°C had no effect on apertures in the absence of ABA, but apertures were reduced for all concentrations of ABA tested. By contrast, incubation at 40°C caused a significant increase in apertures in the absence of ABA and with 10^–9^ M ABA. At higher ABA concentrations, apertures were similar to, or slightly larger than, those observed at 20°C, suggesting a degree of inhibition of ABA sensitivity at 40°C. We also examined the response of barley guard cells to CO_2_ at these three temperatures. Guard cell responses were again temperature-dependent. However, unlike the response to ABA, we observed maximum CO_2_-induced stomatal closure at 20°C, with increasing temperatures causing an increasing degree of inhibition of the CO_2_ response (Figure 5B).

## Discussion

With few exceptions, all of the components of the guard cell signaling network have to date been identified in model species with kidney bean-shaped guard cells, and similar responses have been assumed for graminaceous species containing dumb-bell shaped guard cells. However, there is increasing evidence for species-specific responses to common regulatory cues, driven by different environmental conditions (Prokic et al., 2006; Mori and Murata, 2011; Merilo et al., 2014). It is therefore important to consider signaling in key crop species as well as laboratory models. Given the current concerns over our ability to increase food production in the face of environmental change and to maintain global food security (van Ittersum et al., 2013), it is desirable to establish a robust experimental system for investigating guard cell signaling responses to the multiple environmental stresses currently faced by cereal crops.

The epidermal peel assay has been used to measure stomatal guard cell responses to external stimuli for several decades (Mansfield et al., 1990; Kim et al., 2010; Kollist et al., 2014). Although it has been suggested that the removal of the stomatal complex from the biochemical and physical influences of the mesophyll tissues means that the epidermal peel assay cannot always accurately reflect stomatal responses in intact leaves (Lee and Bowling, 1992; Roelfsema and Hedrich, 2002), it has nevertheless served as an important tool in the elucidation of the complex signaling network within guard cells (Mansfield et al., 1990; Hetherington and Brownlee, 2004; Kim et al., 2010). Despite this fact, few studies have examined the molecular mechanisms by which the dumb-bell shaped guard cells of the Gramineae respond to environmental stimuli and whether these reflect our current understanding of the signaling network in the kidney bean-shaped guard cells of the model species studied to date. This has been due largely to graminaceous species being considered intractable to the necessary cell physiological techniques. We have demonstrated here that the isolation of epidermis containing viable guard cells, whilst technically more demanding than in other model species, can be established as a routine technique to permit such investigations.

Epidermis was most easily obtained from young plants and guard cell viability was capable of being maintained at levels suitable for collection of aperture data from large numbers of stomata. While in all three species tested there was significant variability between individual peels, overall guard cell viability was maintained at around 70–80% for 4 h in barley and *Brachypodium*, although it declined to around 40% in wheat. This compares to between 85% (*Pisum sativum*) and 100% (*C. communis* and *V. faba*), respectively (Weyers and Travis, 1981). All three species exhibited stomatal closure in response to ABA and CO_2_, consistent with previous reports of ABA- and CO_2_-induced stomatal closure in isolated epidermis of model species with kidney bean-shaped guard cells (for reviews, see Hetherington and Brownlee, 2004; Kim et al., 2010; Mori and Murata, 2011). These responses highlight the epidermal peel assay as a useful tool for dissecting guard cell signaling pathways in grasses.

We used the epidermal peel assay to measure barley guard cell responses to ABA, CO_2_ and temperature alone and in combination. In experiments where we simultaneously applied two closing signals, ABA and CO_2_, we observed a simple additive response at 20°C, whereby apertures were smaller in the presence of both ABA and CO_2_ than for the individual stimuli. Interactions between either ABA or CO_2_ and temperature, however, were more complex. Incubation of epidermal strips under opening conditions (light, CO_2_-free air, no ABA) at different temperatures resulted in similar apertures, but with a small but statistically significant increase in aperture at 40°C. Upon addition of ABA, apertures were reduced much more markedly at 30°C than at 20°C or at 40°C. The enhanced closure at 30°C could result either from altered biophysical properties of the stomatal complexes, or from increased sensitivity to ABA. Since the effect was only apparent at 30°C, and not at 40°C, a simple biophysical effect of temperature seems less likely, and we therefore suggest that the temperature-dependency of ABA-induced stomatal closure in barley reflects interactions between temperature and ABA signaling pathways. Similar increases in sensitivity to ABA at elevated temperatures have previously been observed in some dicotyledonous species (Honour et al., 1995; Cousson, 2003). Interestingly, the response of the Arabidopsis *RESPONSIVE TO DESSICATION 29A* (*RD29A*) promoter to exogenous ABA was also enhanced at elevated temperature (Xiong et al., 1999), suggesting the possibility of a more general increase in ABA sensitivity at elevated temperature.

Temperature had a different and very pronounced effect on the response of barley guard cells to external CO_2_. At 20°C, both ambient and elevated CO_2_ treatments resulted in a substantial reduction in stomatal aperture. At 30°C, the effect of ambient CO_2_ was strongly diminished, and whilst guard cells still responded to elevated CO_2_ at 30°C, responses to both ambient and elevated CO_2_ concentrations were lost at 40°C. These observations are consistent with previous work in maize (Raschke, 1970) and bean (Spence et al., 1984), where a loss of stomatal responses to CO_2_ at higher temperatures was found for both species. Since barley guard cells are able to close in response to ABA at 40°C (Figure 4A), an interaction between temperature and CO_2_ signaling pathways again provides the simplest explanation of our data.

Together, our results clearly demonstrate the suitability of the epidermal peel assay for studying guard cell signaling networks in the dumbell-shaped guard cells of the Gramineae and that these studies can provide important insights into the mechanisms by which the stomata of the world’s major cereal crops respond to the multiple stresses resulting from predicted future changes in global temperature, CO_2_ levels and water availability (Stocker et al., 2013). Such studies will help to inform future strategies for improving the water use efficiency of cereal crops and for mitigating the adverse effects of climate change on cereal crop production.

### Conflict of Interest Statement

The authors declare that the research was conducted in the absence of any commercial or financial relationships that could be construed as a potential conflict of interest.
